# Heart regeneration in the salamander relies on macrophage-mediated control of fibroblast activation and the extracellular landscape

**DOI:** 10.1038/s41536-017-0027-y

**Published:** 2017-07-27

**Authors:** J. W. Godwin, R. Debuque, E. Salimova, N. A. Rosenthal

**Affiliations:** 1 The Jackson laboratory, Bar Harbor, ME 04609 USA; 2 Australian Regenerative Medicine Institute, Monash University, Clayton, VIC 3800 Australia; 3 MDI Biological Laboratory, Bar Harbor, ME 04609 USA; 4 National Heart and Lung Institute, Imperial College London, London, UK

## Abstract

In dramatic contrast to the poor repair outcomes for humans and rodent models such as mice, salamanders and some fish species are able to completely regenerate heart tissue following tissue injury, at any life stage. This capacity for complete cardiac repair provides a template for understanding the process of regeneration and for developing strategies to improve human cardiac repair outcomes. Using a cardiac cryo-injury model we show that heart regeneration is dependent on the innate immune system, as macrophage depletion during early time points post-injury results in regeneration failure. In contrast to the transient extracellular matrix that normally accompanies regeneration, this intervention resulted in a permanent, highly cross-linked extracellular matrix scar derived from alternative fibroblast activation and lysyl-oxidase enzyme synthesis. The activation of cardiomyocyte proliferation was not affected by macrophage depletion, indicating that cardiomyocyte replacement is an independent feature of the regenerative process, and is not sufficient to prevent fibrotic progression. These findings highlight the interplay between macrophages and fibroblasts as an important component of cardiac regeneration, and the prevention of fibrosis as a key therapeutic target in the promotion of cardiac repair in mammals.

## Macrophages necessary for heart regeneration

Heart regeneration in salamanders is dependent on the activation of immune cells. James Godwin of The Jackson Laboratory and MDI Biological Laboratory in the US and colleagues depleted all major organs of a group of Mexican salamanders of macrophages, an immune cell responsible for removing cellular debris. They then injured the salamanders’ heart wall with a liquid nitrogen-cooled probe. Unlike adult mammals, zebrafish and salamanders can normally regenerate their hearts after injury. The team found that macrophage-depleted salamanders were unable to regenerate their hearts compared to a control group. Macrophage depletion led to the formation of a permanent fibrotic extracellular matrix scar. But it did not affect the proliferation of heart muscle cells, indicating that their function is not sufficient to prevent the progression of injury toward fibrosis instead of regeneration.

## Introduction

The adult mammalian heart has very limited capacity for repair following major insult. Teleost fish and urodele amphibians (i.e., zebrafish and salamanders) are leading models in vertebrate cardiac regeneration that can regenerate their hearts after amputation of the ventricle apex throughout their lives,^1, 2^ rebuilding a new compact myocardial layer and undergoing full regeneration of up to 20% of organ mass within 2 months. These species respond to tissue loss without scarring, producing new tissue that restores complete function.^2–4^ The ability of the adult fish and salamander heart to replace and re-pattern lost cardiac tissue appears be a capricious evolutionary variable; in the non-urodele amphibian adult, cardiac resection leads to little or no regeneration, leaving fibrosis as the dominant histological reaction to injury.

As neonates, mammals such as the mouse recapitulate the dramatic regenerative response of fish and salamander hearts, but this property is lost shortly after birth,^5^ suggesting that although we retain the necessary genetic programming for cardiac regeneration, other features of the adult mammalian heart intervene to suppress these programs in adulthood. Although our own compromised cardiac regenerative capacity has been attributed largely to the incapacity of adult cardiomyocytes to proliferate after injury and contribute to tissue repair, it is increasingly evident that fibrosis plays a central role in impeding cardiac tissue replacement in the adult mammalian heart.^6–8^ Myocardial infarction (MI), results in necrotic lesions where removal of dead tissue and remodeling in the wound site is not accompanied by tissue regeneration, leading to heart failure and arrhythmias.^9^ Initial inflammation is critical for clearance of tissue debris, stabilization and strengthening of the myocardial wall and prevention of cardiac rupture, yet persistent inflammation leads to scar formation, functional compromise and ultimately heart failure.^6, 10, 11^ Thus the orchestration of the inflammatory response and its resolution is a key factor in effective cardiac regeneration that appears to have been lost both in ontogeny (regeneration of neonatal vs. adult mammalian hearts) and phylogeny (regeneration of fish and salamander vs. mammalian hearts).^12^

The immune response to injury is a major factor controlling tissue repair in a range of mammalian wound healing contexts. In the neonatal mouse heart, which fully recovers from injury,^5^ cardiac regeneration depends on the action of macrophages, as their depletion blocks repair and leads to fibrosis typical of adult mammalian cardiac damage response.^13^ We have previously documented an early requirement of macrophages for the successful limb regeneration in axolotl, a urodele amphibian,^14^ which relies on dedifferentiation of mature tissues in an epimorphic process to form a mound of proliferating cells called a blastema. Muscle cells in the axolotl are formed in the new limb almost exclusively from resident muscle progenitor cells Pax7+ whereas other urodele species (newts) also use the process of myocyte dedifferentiation.^15^ In the axolotl, cardiac regeneration depends on the proliferation of existing cardiomyocytes to replace the damaged myocardium,^16^ but it is unknown whether this process is dependent on immune signaling.

Using the axolotl, we established a cardiac cryo-injury model which induces a necrotic ischemic injury, more accurately recapitulating many hallmarks of clinical ischemia including cardiomyocyte (CM) death and macrovascular reperfusion^9^ compared to the more widely studied resection model. Cryo-injured fish and salamander ventricles regenerate without the involvement of compact scar tissue but form a transient collagenous network that allow activated cardiomyocytes to migrate into and repair the heart muscle wall.^17^ Here, we show that in the adult axolotl, depleting macrophages in the context of cardiac cryo-injury limits fibroblast activation, modifies extracellular matrix (ECM) synthesis, remodelling, and cross-linking profiles, and blocks regeneration despite the continued proliferation of activated cardiomyocytes. These results reveal that tissue regeneration in the heart is dependent on an ECM structure and composition that is compatible with cardiac muscle replacement, and that macrophages may play a critical role in preventing precocious ECM maturation.

## Results

### Necrotic cryo-injuries are regenerated in axolotls

**Fig. 1:**
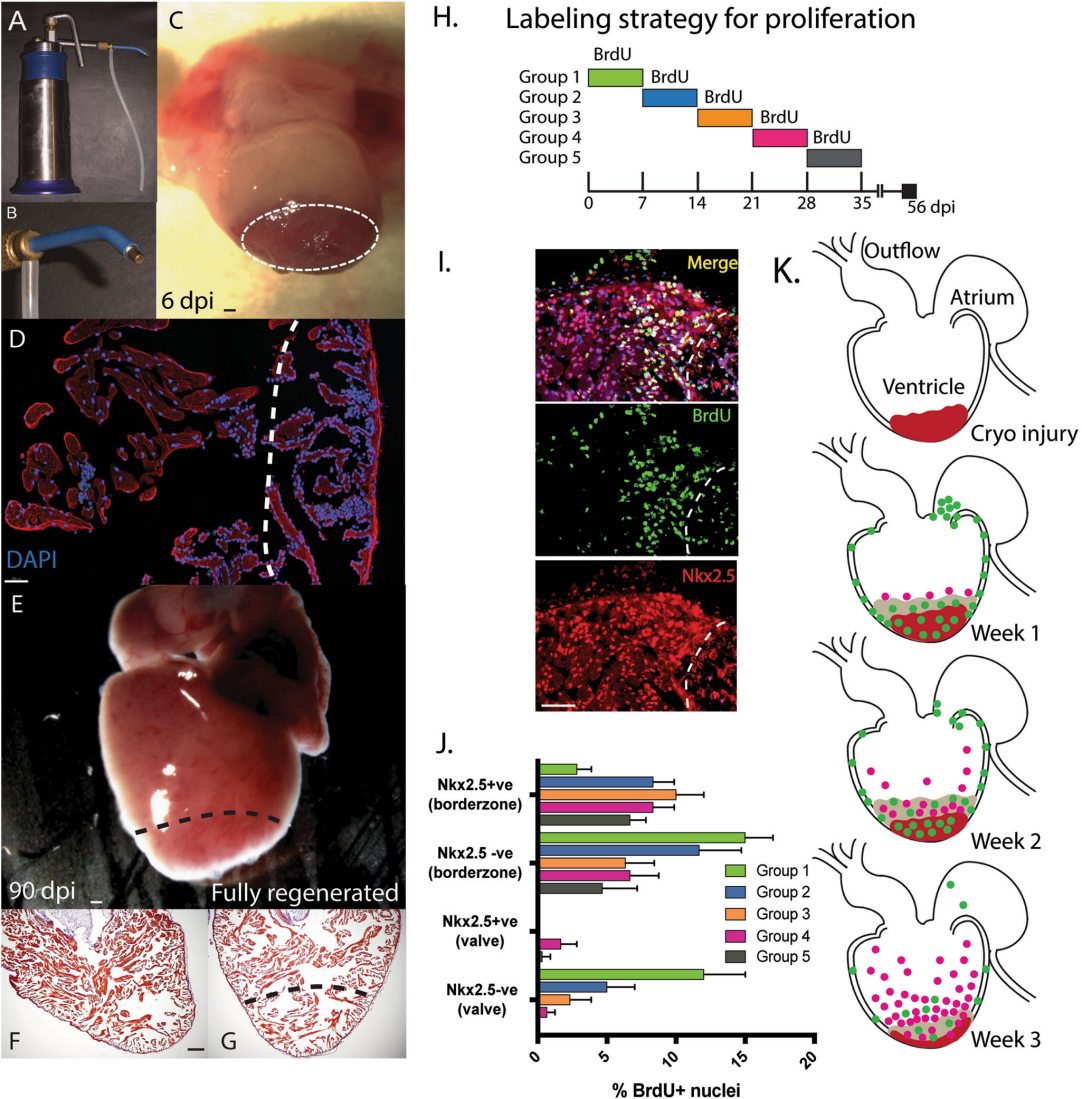
Proliferation of cardiomyocytes during cardiac regeneration post cryo-injury. **a** A commercial liquid nitrogen cooled cryo-gun was used to generate a cryo-lesion at the apex of the heart. **b** Different gold tip attachments allowed matching to animal size. **c** A necrotic lesion was produced with edema still present at 6-days post injury (Dpi). **d** The lesion was identified by a robust inflammatory infiltrate with visible peripheral nuclei (DAPI) at 6 dpi. The ventricle was fully regenerated by 60–90 dpi and appeared normal, both superficially **e**, and histologically **f**, relative to uninjured controls. **g** Histological staining was performed with Acid Fuchsin orange G (AFOG) whrere collagen stains blue, muscle of the myocardium in orange/red and the nucleus black. **h** Time course of Bromodeoxyuridine (BrdU) labelling of animals daily (i.p. injection). **i** Identification of cardiomyocyte (CM) specific proliferation used nuclear localized Nk2.5 antibody staining and dual positive BrdU+ (*Green*)+ Nkx2.5+ cells (*Red*). **j** CM specific and non-CM proliferation was measured during the first 5 weeks of regeneration within 200 μM of the borderzone or 100μM of the valve region (at the junction between the atrium and the ventricle). At least three sections per animal and three animals per time-point were used. **k** Cartoon of the general spatial localization of CM and non-CM proliferation in the axolotl heart over the first 3 weeks of regeneration. Non-cardiomyocytes shown in *green* and Nkx2.5+ CM shown in pink. Cryo lesion in ventricle shown in *red*. Scale bar = 100 μM

Damage to the heart ventricle by puncture or resection in both newt and axolotl salamanders results in transient ECM synthesis and functional replacement of missing or damaged cardiomyocytes, epicardium, endothelium and connective tissue cells.^16, 18, 19^ In an alternate approach similar to that developed in mice and zebrafish^9, 17, 20^ we used a liquid nitrogen cooled gold probe to reproducibly induce targeted tissue damage on the ventricle wall in order to monitor healing and repair over the time course of regeneration **(**Fig. 1a, b **)**. The cryo-lesion resulted in tissue damage and necrosis **(**Fig. 1c **)**, requiring debridement and cell replacement. Robust infiltration of nucleated cells was consistent with invasion of inflammatory leukocytes **(**Fig. 1d). Cryo-injuries were completely repaired by 60–90 days **(**Fig. 1e–g **)** similar to that reported for ventricular resection.^16^

### Regeneration relies on cardiomyocyte proliferation in a temporal sequence of activation

Heart regeneration in the salamander involves the activation of cardiomyocyte proliferation. We assessed the time-course for proliferation in the cryo-injured heart by assessing Bromodeoxyuridine (BrdU) incorporation in both cardiomyocytes (Nkx2.5+ve) vs. non-cardiomyocyte (Nkx2.5−ve) populations in each week following cardiac cryo-injury **(**Fig. 1h–j). A distinct sequence for proliferation was observed, starting with epicardial activation and a burst of proliferation in the valve region in week 1 (Fig. 1k). As seen in fish cardiac injury response,^21^ epicardial proliferation was observed globally and not limited to the injury site. In week 2, cardiomyocyte proliferation was increased and mainly restricted to the border zone of the injury site, accompanied by continued cell proliferation in the epicardium. In week 3, cardiomyoyte proliferation peaked in a dense layer around the injury border zone with a scattering of cardiomyocyte proliferation extending into remote regions of the ventricle. Proliferation continued to decline until regeneration was complete (Fig. 1j).

### Macrophage depletion prior to cardiac cryo-injury results in impaired cardiac function and poor survival

**Fig. 2:**
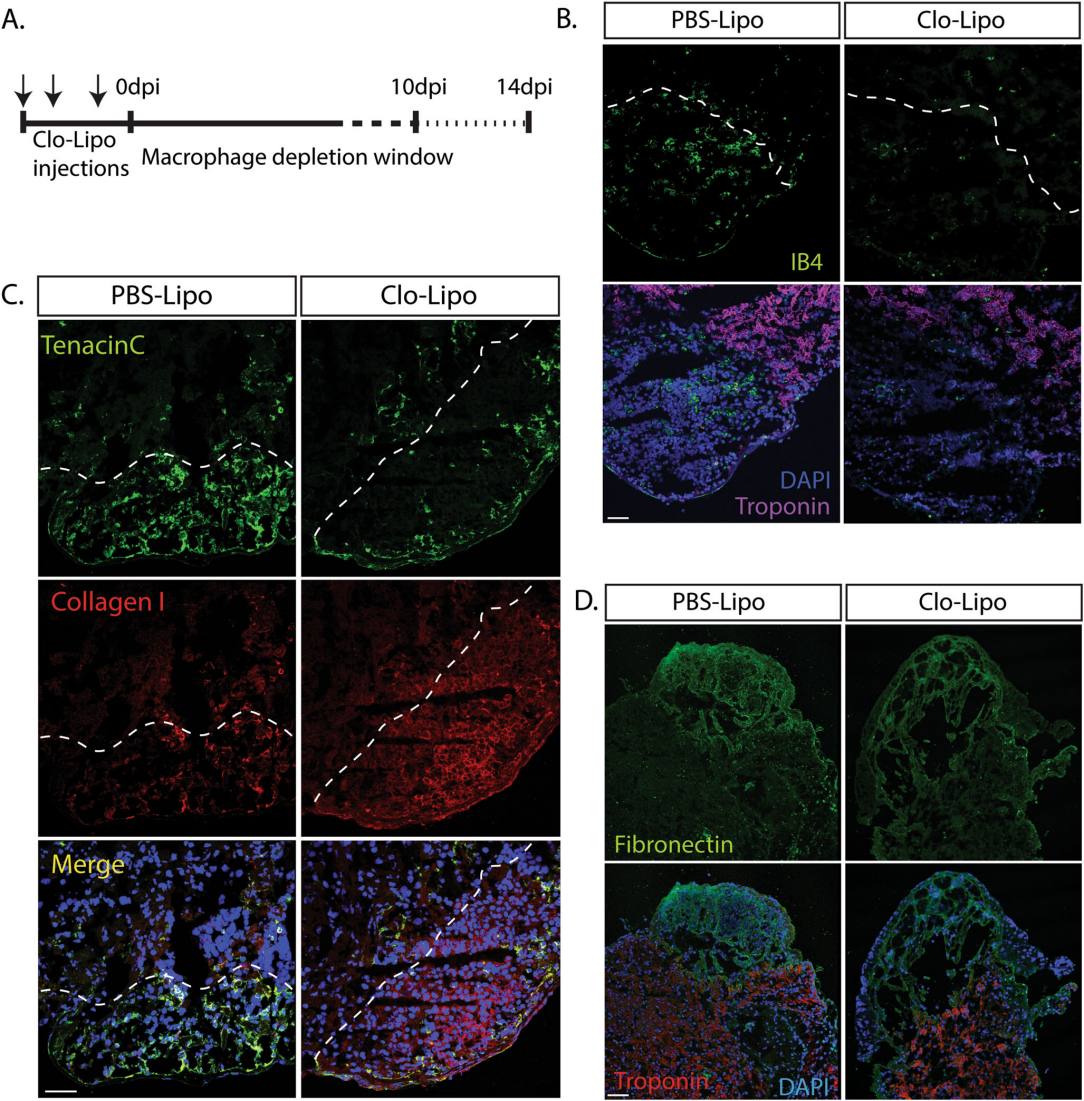
ECM deposition during the early healing phase of cardiac regeneration is altered in macrophage-depleted animals. **a** Macrophage depletion strategy using Clodronate liposomes (Clo-Lipo) with a triple injection prior to cryo-injury. **b** Cardiac troponin T staining was absent within the cryo lesion at 7 dpi. Clo-Lipo treated animals showed almost complete depletion of macrophages (marked by Isolectin B4) in the cryo-injured heart at 7 dpi relative to PBS-Lipo control animals. **c**–**d** Critical components of the transient extracellular matrix (ECM) were dysregulated in Clo-Lipo treated animals at 7 dpi relative to PBS-Lipo control animals. Collagen I, was up-regulated and muted expression of Tenascin C and fibronectin is observed. Scale bar = 100 μM

To test the dependence of adult axolotl heart regeneration on macrophage function we employed a clodronate loaded liposome (Clo-Lipo) macrophage depletion regimen that eliminates macrophages from all major organs after three *i.v*. injections over 4 days.^14^ This procedure depletes resident macrophages from liver and spleen due to access via the fenestrated vasculature. Although the depletion of cardiac resident macrophages may not be absolute, the enhanced trabeculation of the salamander heart, relative to the mouse, does provide enhanced access of clodornate liposomes to resident macrophages. Axolotl hearts were cryo-injured during a Clo-Lipo macrophage-depleted window of around 7–10 days before macrophage repopulation arises from myelopoiesis **(**Fig. 2a, Supp. Fig. 1A **)**. Cryo-lesions were located by the absence of cardiac troponin T within the ventricle, and reduction in macrophage accumulation was identified by the specific macrophage marker Isolectin-B4^22^ (Fig. 2b, Supp. Fig. 1A). Clo-Lipo macrophage depletion altered the production of key ECM proteins (Tenascin C, Collagen-I and Fibronectin) within the lesion site when compared with the phosphate buffered saline loaded liposome (PBS-Lipo) control animals **(**Fig. 2c, d, Supp. Fig. 1B).

**Fig. 3:**
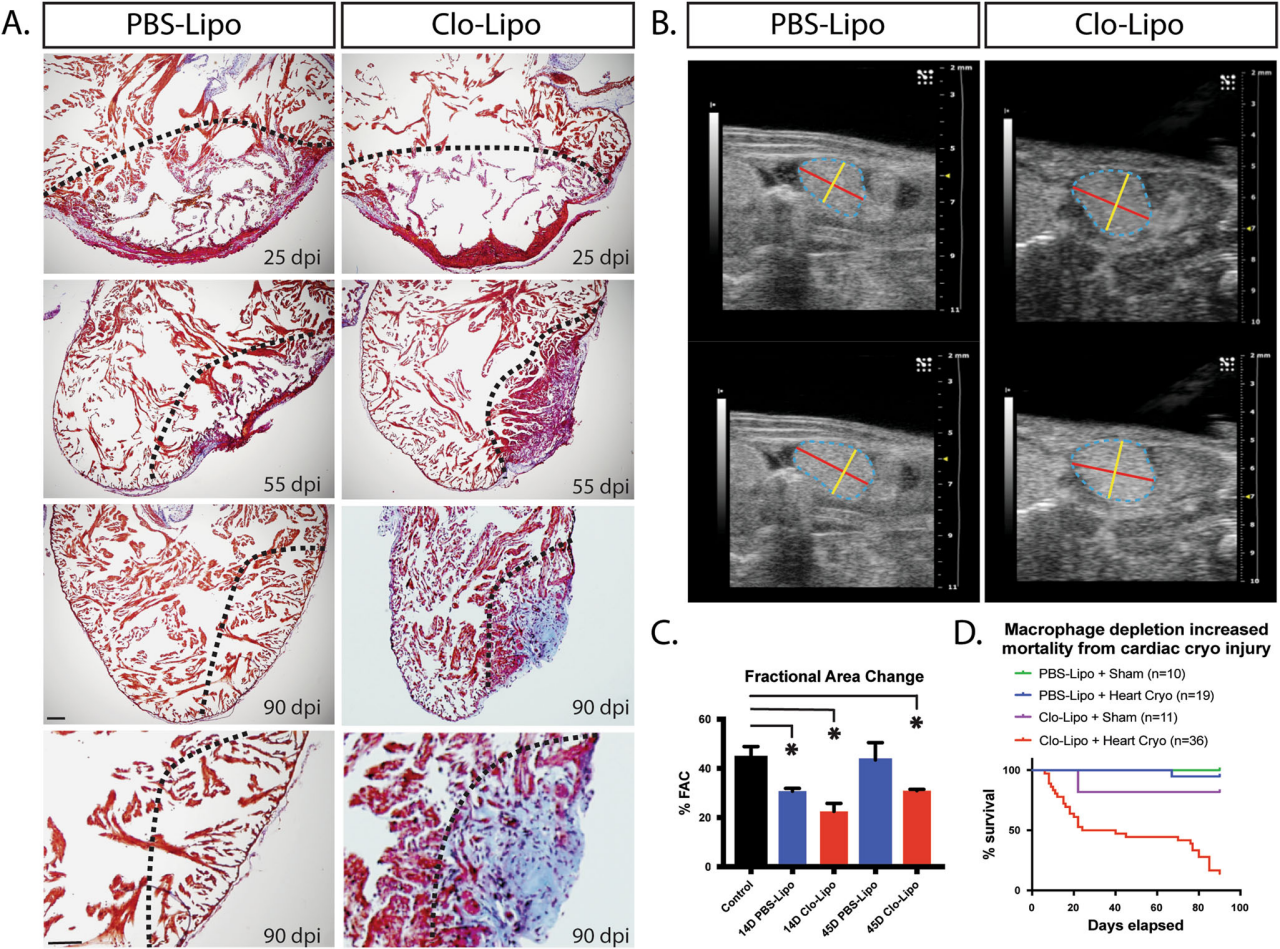
Macrophage depletion results in progressive scar formation and regenerative failure. **a** AFOG staining of Clo-Lipo macrophage depleted animals vs. PBS-Lipo control animals over the normal time-course of regeneration. Collagen deposition (*blue*) and fibrosis developed in the absence of macrophages and was not remodeled. **b** Ultrasound examination of the hearts post cryo-injury in macrophage depleted and normal animals. Representative end systolic (*top*) and end diastolic (*bottom*) images of the ventricles in the maximal longitudinal plane. Trace of the ventricle is shown in dashed blue line. Diameter marked with *yellow* and length with *red* overlays. **c** Assessment of cardiac performance based on calculations of % Fractional Area Change (%FAC). Cryoinjury results in a significant %FAC reduction in both Clo-Lipo and PBS-Lipo treated animals relative to uninjured control at 14 dpi. PBS-Lipo control animals regained normal function by 45 dpi while Clo-Lipo animal hearts remained compromised. **d** Clo-Lipo treatment altered animal survival post-cryo-surgery in two major phases. Scale bar = 500 μM

The effect of cryo-injuring the axolotl heart in the absence of macrophages was dramatic, with progressive scarring occurring in macrophage depleted hearts evident at 25, 50, and 90-days post-injury (Fig. 3a), accompanied by a change in ventricular function assessed by ultrasound analysis **(**Fig. 3b **)**. Fractional Area Change (FAC) was used as a functional parameter. %FAC was significantly reduced at 14 days relative to uninjured in both PBS injected animals and Clo-Lipo treated animals **(**Fig. 3c **)**. The PBS-Lipo treated animals showed functional recovery by 45 days whereas Clo-Lipo treated animals showed reduction in functional improvement at 45-days relative to uninjured and PBS injected animals. Similar results were obtained using independent LV(LeftVentricular)-trace mode that allows for volumetric calculations (e.g., %EF and stroke volume) calculations (Data not shown). Long-term animal survival was also severely impacted in Clo-Lipo treated animals despite restoration of macrophage function around 10-days post-injury **(**Fig. 3d). This mortality was not observed in limb regeneration model using the same Clo-Lipo regimen.^14^ Macrophage depletion placed the short-term survival at risk from infection post injury, however the impacts on long-term survival were likely related to heart failure as no signs of infection were observed in these animals.

### Macrophage depletion results in disruption of the regeneration program by induction of fibroblast activation and alternative ECM profile

**Fig. 4:**
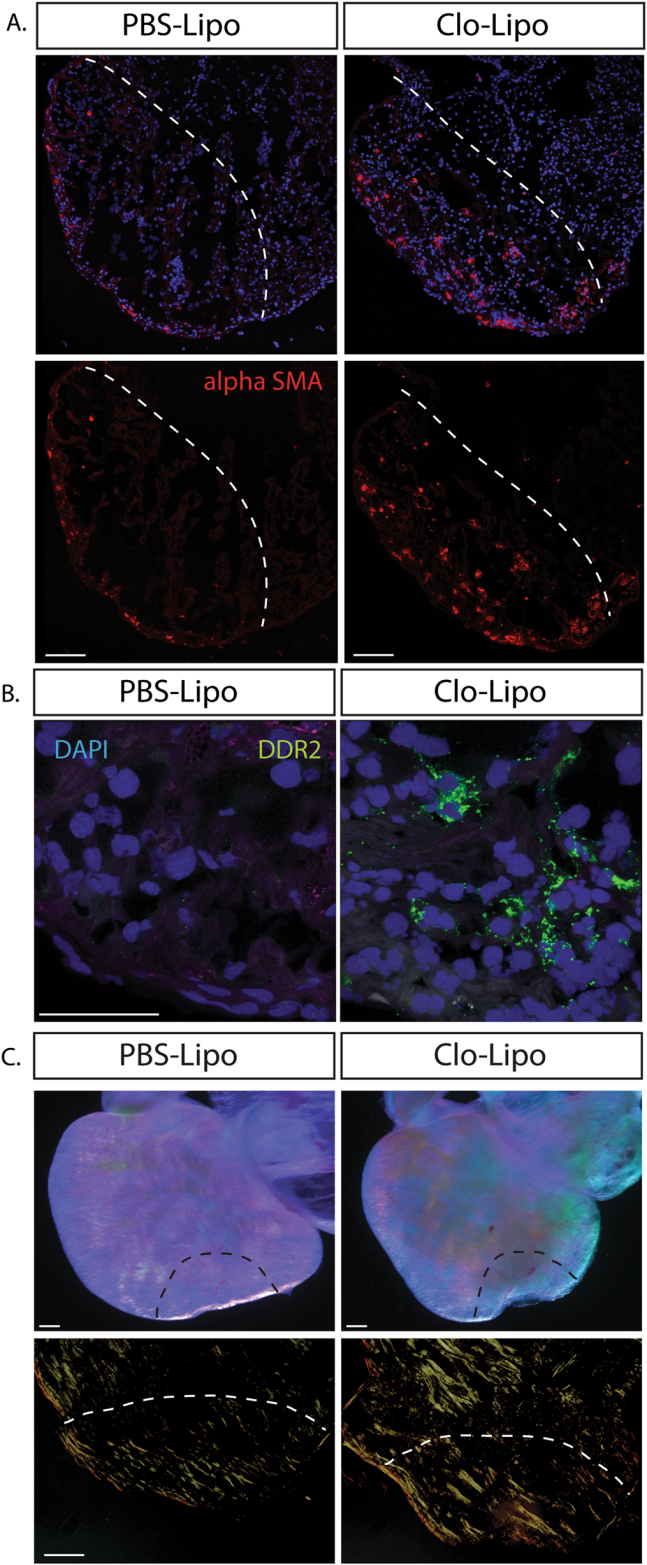
Macrophage depleted animals feature enhanced fibroblast differentiation and collagen maturation. **a** Alpha smooth muscle actin (αSMA) positive myofibroblasts populated the cryo-lesion in Clo-Lipo treated animals where αSMA + cells were restricted to the epicardium in PBS-Lipo control lesioned animals at 7 dpi. **b** Discoidin Domain Receptor Tyrosine Kinase 2 (DDR2) expression was up-regulated in Clo-Lipo treated animals 7 dpi at different sites at along the wound margin. **c** Dissected 14 dpi cryo-injured whole mounted hearts imaged under a polarizing lens at maximum contrast. Clo-Lipo hearts showed enhanced accumulation of cross-linked collagen (top panel) Picrosirius red stained sections visualized under polarized light showed accumulation of thick collagen bundles in Clo-Lipo treated animals at 14 dpi. Scale bar = 100 μM

To determine the processes leading to the dramatic scar tissue accumulation and lack of collagen remodeling back to normal tissue in macrophage-depleted hearts, we first characterized ECM components that were up-regulated following cryo-injury in both PBS-Lipo control animals and macrophage depleted Clo-Lipo animals. The region of cryo-injury was determined by the depletion of cardiac troponin T staining and sarcomeric myosin in injured cardiomyocytes (see Fig. 2b). Using this landmark, we assessed that fibronectin was specifically up-regulated within the lesion site in control animals but a muted response is observed in macrophage depleted animals **(**Fig. 2d), accompanied by robust induction of Alpha-Smooth Muscle Actin (αSMA) positive cells within the lesion **(**Fig. 4a Supp. Fig. 1A **)**. In PBS-Lipo control animals (αSMA) positive cells were confined to the epithelial region and were absent within the lesion. Discoidin domain receptor 2 (DDR2) positive cells were also present specifically in Clo-Lipo treated animals **(**Fig. 4b, Supp. Fig. 1A **)**. These represent highly conserved and specific markers for activated subsets of fibroblasts associated with the promotion of collagen synthesis and fibrosis.^23^ Macrophage depletion also produced changes in collagen deposition and arrangement as measured by picosirius red and polarized light, consistent with the limb regeneration model **(**Fig. 4c **)**.

**Fig. 5:**
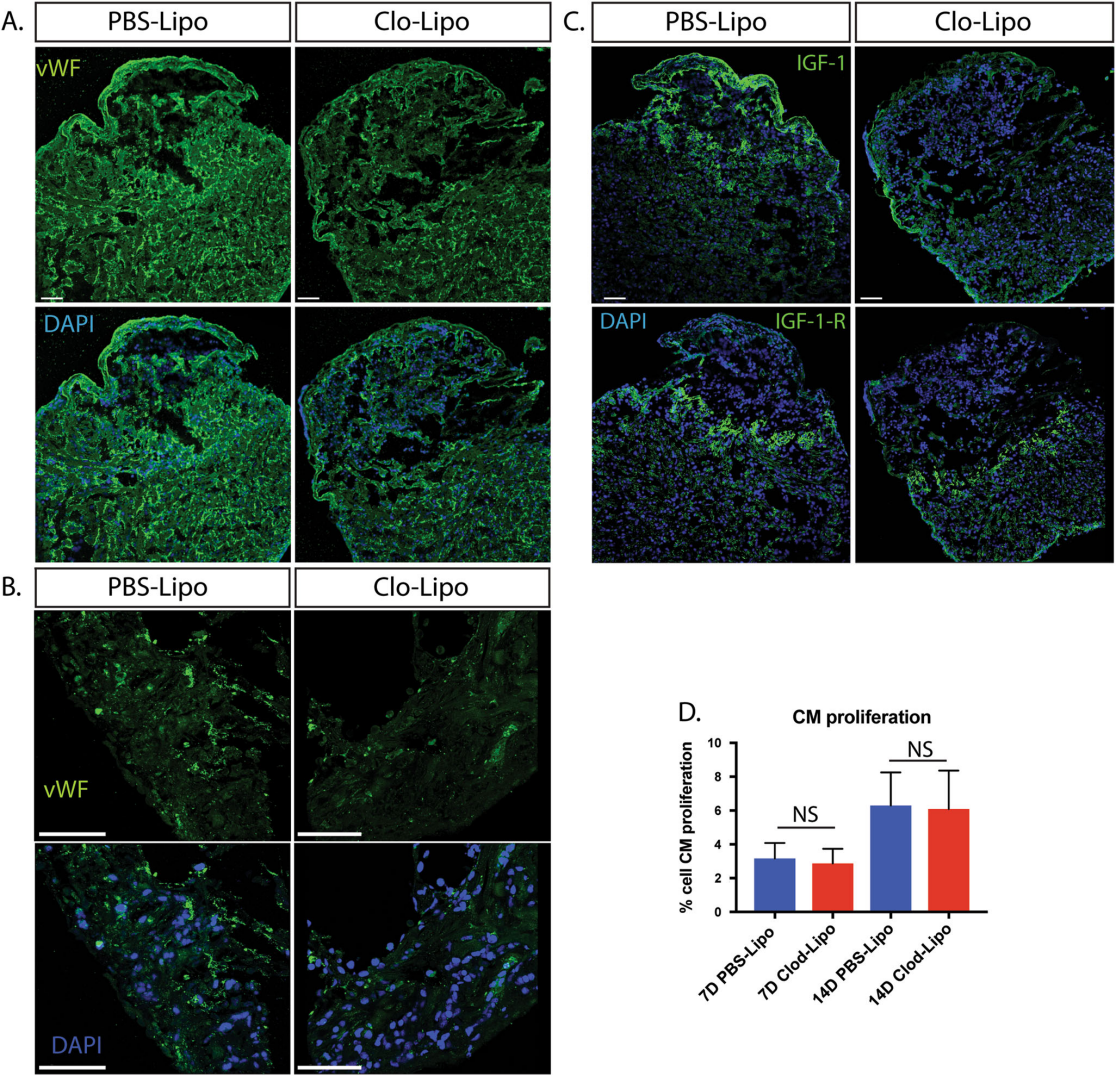
Blood vessel density and IGF signaling in macrophage depleted animals is altered in Clo-Lipo animals where cardiomyocyte proliferation appears unchanged. **a** Endothelial cell staining using von Wilebrand Factor (vWF) demonstrated a reduced vascular network in Clo-Lipo animals at 14 dpi. **b** higher magnification of vWF staining at wound margin showed reduction in blood vessel outgrowth. **c** Insulin-like Growth Factor (IGF-1) ligand expression was downregulated in Clo-Lipo injured hearts at 14 dpi with weak epicardial expression maintained. Conversely, IGF-1-Receptor expression was upregulated specifically in cardiomyocytes (CMs) at the wound margin and maintained in Clo-Lipo treated animals. **d** CM proliferation was measured using PCNA+ Nkx2.5+ cells on 7 and 15 dpi hearts and no significant difference was detected. CM proliferation measured using at least three sections per animal and three animals per time-point. Scale bar = 100 μM

Macrophages play an important role in regulating new blood vessel and lymphatic vessel growth in many species. To determine whether the lack of macrophages during the early phase of injury affected vessel sprouting at 14 days post injury, we stained von Wilebrand factor (vWF) positive endothelial cells to reveal blood vessel sprouting Fig. 5a–b **)** maintained at a low level within the cryo-lesion wound margins of Clo-Lipo animals, but vessel density was severely reduced relative to PBS-Lipo treated animals (Supp. Fig. 1B **)**.

Insulin-like growth factor 1 (IGF-1) is a major driver of heart repair and some isoforms are produced by macrophages.^24^ Expression of the IGF-1 ligand was weakly maintained in the epicardium but was noticeably absent in the remainder of the lesion in macrophage-depleted animals **(**Fig. 5c, Supp. Fig. 1B **)**. Interestingly, the IGF1 Receptor was upregulated in cardiomyocytes at the wound margin in both macrophage-depleted and control hearts (Fig. 5c, Supp. Fig. 1B **)**, indicating that the failure in regeneration is not likely due to a lack of cardiomyocyte responsiveness to IGF-1.

### Axolotl cardiomyocyte proliferation is maintained despite regenerative failure

The lack of sufficient cardiomyocyte proliferation in mammals is generally considered to be a major obstacle to regeneration. To determine whether macrophage depletion directly affects CM replenishment by potentially limiting growth factors or cytokine stimulation, we measured CM proliferation at 7 and 14-days post-injury in macrophage-depleted axolotl hearts using proliferating cell nuclear antigen (PCNA) staining of NKx2.5+ cardiomyocytes **(**Supp. Fig. 2). These time-points span the period before and during the initial wave of macrophage replenishment. We found no significant difference in CM proliferation at these time points **(**Fig. 5d). The activation of CM proliferation in the absence of macrophages indicates that CM deficiency is not the cause of regenerative failure in macrophage-depleted cryo-injured animals.

### Fibroblast activation and enzymatic ECM crosslinking causes regenerative failure in the axolotl

**Fig. 6:**
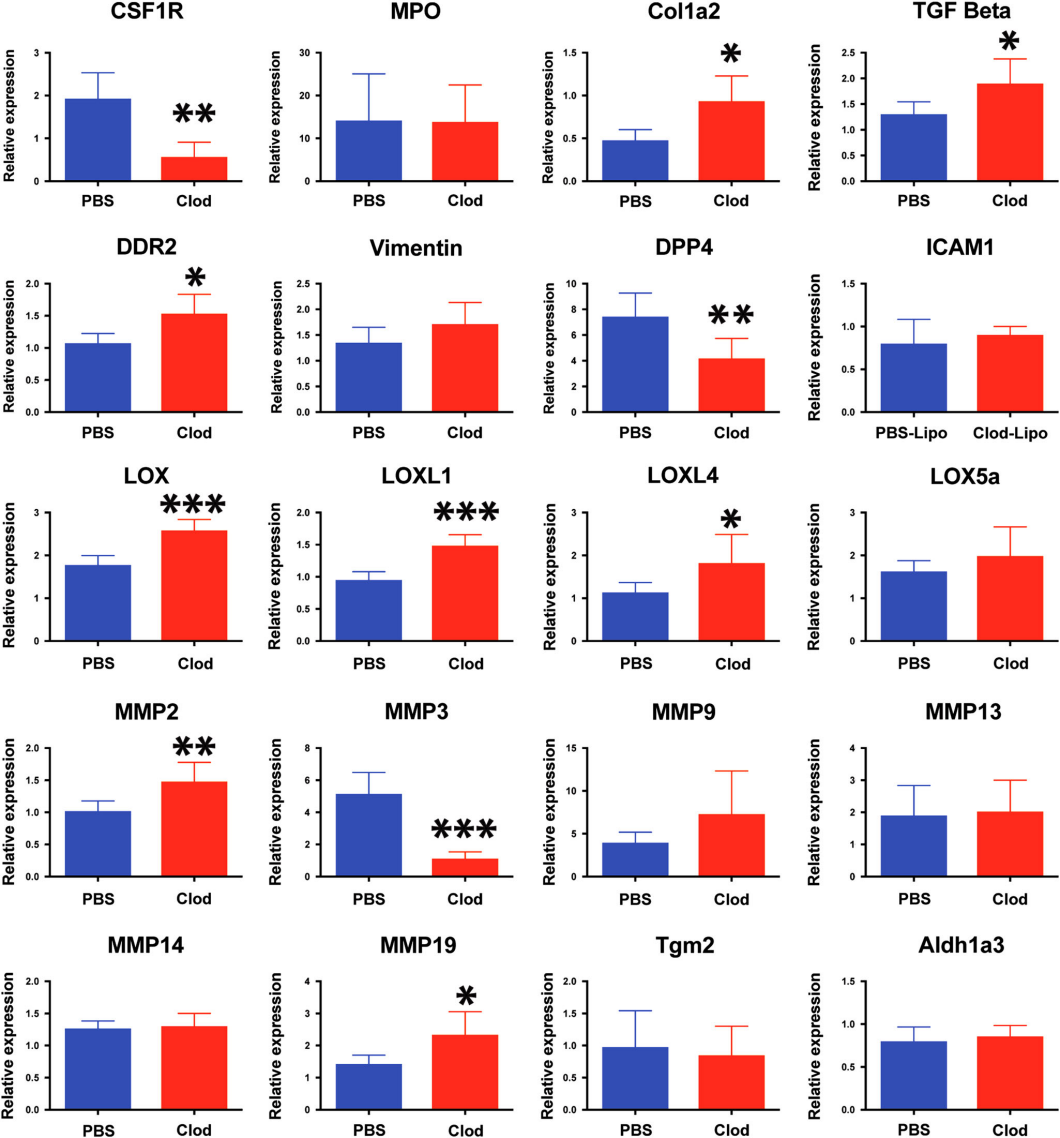
Gene expression analysis of macrophage depleted hearts post cryo-injury identifies molecular targets regulating ECM regulation during regeneration. RT-PCR analysis of 7 dpi cryo-injured hearts isolated from Clo-Lipo and PBS-Lipo control animals. Heart ventricle samples were screened for a list of candidate genes (partially isolated from previous RNAseq studies) that regulate ECM turnover and maturation during regeneration. *N* = 5 animals, at least 3 independent experiments performed. (see Methods section for statistics)

To determine a possible mechanism whereby transient ECM deposition could be converted to a permanent scar, molecular analysis was conducted on the heart ventricle of both control and macrophage-depleted animals. Colony stimulating factor 1 receptor gene expression confirmed efficient depletion of macrophages where myeloperoxidase gene was maintained indicating a normal neutrophil response. (Fig. 6 **)**. Discoidin Domain Receptor Tyrosine Kinase 2 (DDR2) and Collagen-I up-regulation was detectable in the Clo-Lipo treated animals confirming immuno-histochemical analysis. Vimentin marks all fibroblasts in the axolotl and the vimentin transcriptional signal indicated that the number of fibroblasts in the heart was not significantly affected. Post-injury, Dipeptidyl peptidase-4 was downregulated in macrophage-depleted lesions, implicating a conserved role as a macrophage-dependent protease in wound remodeling. Transforming growth factor beta (TGFβ) expression was elevated in macrophage-depleted animals, whereas Matrix metaloproteinase (MMP) enzymes MMP2, MMP19 were upregulated, MMP3 was significantly down-regulated and MMP9 was not significantly changed. This implicates MMP3 as the major macrophage-dependent MMP, while expression from neutrophils may compensate for other MMPs. The molecular pathways responsible for the highly cross-linked ECM phenotype likely include a gene group belonging to the Lysl-oxidase enzyme family (LOX) involved in collagen crosslinking and tissue maturation, which showed high dysregulation across several family members, while other pathways that were not affected included Transglutaminase 2 involved in fibrosis, and Aldehyde Dehydrogenase 3 Family Member A2 involved in retinoic acid synthesis (Fig. 6).

## Discussion

This study highlights the central role of fibrotic pathways in limiting efficient cardiac repair, and demonstrates an immediate requirement for macrophages in regeneration of adult cardiac tissue, as their ablation at early time-points has lasting effects on the outcome of the cardiac injury response: progressive fibrosis results in defective cardiac regeneration and abrogates functional recovery. The detection of activated fibroblasts or myofibroblasts at very early stages of repair in macrophage-depleted animals indicates that macrophages are immediately required to control fibroblast differentiation. Whereas early macrophage depletion could be expected to affect viability and recovery from surgery due to associated risk of infection, animals surviving this initial period are at increased risk at much later time points, coinciding with progressive fibrosis leading to terminal heart failure. These results are reminiscent of mammalian MI injury where dilation and fibrotic progression ultimately destroys heart function,^25^ and are consistent with a model inducing unresolved inflammation in the regenerating amphibian limb, where cellular dedifferentiation and reprogramming still occur locally, but the tissue interactions and signaling required for limb patterning cannot be established, leading to regenerative failure.^26^

In the hearts of macrophage-depleted cryo-injured axolotls, initiation of CM proliferation was unaffected at 7 and 14-days post-injury. The fact that the CM response was largely intact in hearts damaged by the absence of macrophages indicates that CM proliferation is not the limiting step in axolotl heart regeneration. Cardiomyocytes at the lesion site functionally responded to injury by upregulating IGF1R at the borderzone and although IGF-1 expression was reduced in clodronate-induced cryo-lesions, it was partially maintained in the epicardium, a potent source of growth factor signaling in the zebrafish.^27^ It is therefore possible that epicardially derived growth factors are sufficient to maintain CM proliferation in the cro-injured axolotl heart.

Macrophages play an essential role in many injury contexts by supporting angiogenesis through expression of Vascular endothleial growth factor A.^28, 29^ We observed changes in vWF+ endothelial populations between macrophage depleted and control cryo-lesions. This supports a role for macrophage-dependent angiogenesis although it is not known if the new blood vessel growth arises from sprouting or from epicardial derived endothelial progenitors in this model.^30^ At 10 days after clodronate treatment, macrophage populations are largely restored in axolotls, at which point any pro-angiogenic functions are likely impacted by rigid fibrotic ECM associated with early macrophage depletion. New blood vessel growth is likely to impact the speed of regeneration in axolotls however the thin trabeculated (non-compacted) structure of the myocardium likely allows some access of oxygenated blood to the injured tissue. Therefore is is possible that angiogenisis may not be an absolute requirement for allowing the regeneration of an amphibian heart. Future studies should be aimed at testing the requirement for neoangiogenesis in a non-fibrotic alternative model.

The lack of macrophages in early cryo-lesions does not result in cardiac rupture but alters the ECM landscape, presumably through the activation state of attending connective tissue cells. The progressive nature of fibrosis that appears with cryo-lesions in clodronate-treated animals presumably stems from the early activation of myofibroblasts within the wound and the downstream accumulation of collagen deposition. The differentiation of fibroblasts and other precursor cells into myofibroblast populations is a major driver of the fibrotic response in humans and other mammals.^31–33^ Normal regeneration activates myofibroblast conversion in the epicardium but the depletion of macrophages results in fibroblast activation (alpha SMA+ cells) not normally found within the lesioned site. This observation gives weight to the notion that wound macrophages act as a negative regulator of fibroblast differentiation. In this study it is unclear the exact origin of the participating macrophages and if regulatory function is restrictied to a particular subpopulation. In mammals, myofibroblasts are derived from a range of progenitors.^34, 35^ It is not known if these cell types can act as a source of myofibroblasts in salamander tissues or how the lack of macrophages may enhance fibroblast differentiation.

The histological identification of DDR2+ fibroblasts in macrophage-depleted cryo-lesions, confirmed by Real time PCR (RT-PCR), suggests that at least a portion of fibroblasts in these wounds have altered gene expression. DDR2 is a receptor tyrosine kinase implicated in the regulation of growth, migration, cell morphology, and differentiation and remodeling of the ECM by controlling matrix metalloproteinases.^23^ In mammals, DDR2 is a marker of mesenchymal cells ^36^ and is restricted to cardiac fibroblasts in the adult heart.^37^ In the mouse, DDR2 expression has been linked with cardiac fibrosis through multiple pathways,^23, 38^ as the extracellular discoidin domain of DDR2 binds with high affinity to immobilized collagen type I.^39^ It is therefore likely that DDR2 is an evolutionarily conserved marker for alternatively activated fibroblasts in the salamander heart. Mesenchymal cells are endowed with the remarkable ability to respond to environmental signals triggered by injury or infection and phenotypic states, analogous to the macrophage polarization paradigm.^40^ Dynamic regulatory feedback mechanisms between mesenchymal and macrophage cell types are now appreciated to be important for regulating inflammation and fibrotic activation in a range of contexts.^41^

Cardiac regeneration in salamanders and zebrafish involves the formation of transient ECM deposition that is distinct from normal tissue^18, 42^ and is remodeled in coordination with effective tissue replacement.^43^ Although the fibrotic process is extremely complex due to the dynamic regulation, multiple targets and co-expression of inhibitors that limit function in certain contexts,^44^ a range of candidate genes involved with precocious ECM maturation were identified, including MMP3, the major MMP absent in macrophage depleted animals, and MMP2 and MMP19, which were overexpressed. MMPs have both inhibitory and stimulatory roles in fibrosis that vary by tissue and wound healing phase.^45^ In vitro, MMP3 activates β-catenin signaling and induces epithelial-to-mesenchymal (EMT) transition.^46^ Reduced MMP3 in macrophage-depleted cryo injured hearts may therefore affect EMT, abrogating β-catenin signaling.^47^ MMP2 has been implicated in cardiac fibrosis, associated with cardiac fibroblasts and myofibroblasts rather than inflammatory leukocytes post-cardiac injury.^42, 48^ MMP19 overexpression promotes fibrosis at both early injury and late resolution stages in liver injury where in lung it may play an anti-fibrotic role.^44^

TGFβ is a potent activator of myofibroblast induction in many species and a major regulator of fibrosis in many contexts including the heart.^49^ TGFβ is essential for limb regeneration in the salamander^50^ and it is likely that strict temporal expression is required to maintain functional regeneration circuits. The upregulation of cardiac TGFβ in the absence of macrophages is in agreement with results obtained previously in the limb regeneration model (Godwin *et al*., PNAS 2014). The source of increased TGFβ production in the absence of macrophages is yet to be determined but highlights a role for macrophage-specific cytokine regulation of TGFβ expression in salamander regeneration.

Our analysis uncovered a potential role for the LOX family of enzymes in the precocious maturation of the transient regenerative ECM, as several members of the LOX enzyme family were dysregulated in cardiac cryo-lesions in macrophage-depleted animals. LOX enzymes play an important role in the maturation of ECM proteins and correct assembly of tissue architecture, forming cross links between ECM proteins that stabilize ECM tissues during normal homeostasis.^51^ The early activation of Lysyl oxidase (LOX) and lysl oxidase like−1 (LoxL1), and lysyl oxidase like -4 (LOXL4) but not lysyl oxidase 5a (LOX5a), in the macrophage-depleted axolotl heart post-cryo-injury suggests that macrophage signaling is required to prevent early maturation of the transient ECM required for regeneration. LOXL1 regulates collagen cross-linking and total collagen levels in angiotensin II-induced hypertension in rodents,^52^ has been associated with renal fibrosis^53^ When overexpressed, it induces cardiac hypertrophy in mice.^54^ LOX enzymes have also been implicated in activation of TGFβ1 signaling from the latent complex.^55^ LOXs also contribute significantly to the detrimental effects of cardiac remodeling in mice following MI^56^ and as such may represent a common pathway of fibrotic disruption of repair in a range of evolutionarily distinct species, Inhibition of LOX signaling may therefore be an important therapeutic avenue for limiting fibrosis. Notably, transglutaminase pathways were unaffected by macrophage depletion. Transglutaminase is reported to promote tissue fibrosis through cross-linking extracellular collagen and fibronectin, making them more resistant to breakdown^57^ along with multiple other functions that may promote fibrosis.^58^ The lack of transglutaminase activation may be another key feature of effective cardiac regeneration.

Previous studies of cardiac repair in mouse models have been focused on driving cardiomyocyte proliferation to improve cardiac regeneration (reviewed in refs. 59–61) In the salamander CM proliferation is necessary but not sufficient to drive effective regeneration, suggesting that efforts should be refocused on understanding the dominant fibroblast signals regulating repair. The excessive activation of fibroblasts have been implicated as major determinants of maladaptive repair in a range of mammalian contexts^41^ and the injury induced changes in mesenchymal cell activation appears evolutionarily conserved. Identifying the mechanism by which macrophage-fibroblast interactions repress fibrotic activation in the salamander heart may lead to new molecular targets useful in promoting cardiac regeneration in humans.

## Materials and methods

### Animal maintenance and wounding

Axolotl (*Ambystoma mexicanum*) larvae were maintained in 20% Holtfreter’s solution at 19–22°C on a 12-h light, 12-h dark cycle. Before cryo-injury, animals were anesthetized in 0.1% MS-222 (ethyl 3-aminobenzoate methanesulfonate salt; Sigma-Aldrich, St. Louis, MO). All animals used were at least 1 year old and measured between 4–10 cm for immunofluorescence and Echocardiography and RT PCR. 17–20 cm animals were used for BrdU experiments and Trichrome staining. Animals were randomly assorted into each group without regard to gender with sample sizes chosen assuming an equal variance, a power value of 0.8 and alpha of 0.05. All animal care protocols and methods were performed in accordance with relevant regulations and guidelines and with approval from either the Monash University animal ethics committee or MDIBL animal care and use committee.

### Mononuclear phagocyte depletion

Clodronate liposomes and PBS liposomes (control) preparations were purchased from Encapsula Nano Sciences, (Nashville TN, USA). Method used were described previously.^14^ Maximal depletion protocol = (96 + 48 + 24 h prior to analysis) triple injection protocol (3× *i.v*. injections over 4 days, prior to injury).

### Cryo injury procedure

Animals were anesthetized in 0.05–0.1% MS-222 (ethyl 3-aminobenzoate methanesulfonate salt; Sigma-Aldrich, St. Louis, MO) and positioned on moist kimwipes (kimtech) in a supine positon. Skin was cleaned with chlorohexidine and the heart accessed by keyhole surgery. Briefly, a small incision was made on the skin and thoracic muscle. Using sterile fine forceps and iredectomy scissors, the pericardial sac was opened to reveal the beating heart. A commercial gold probe (Brymill AC-3 with different sized tip attachments) was cooled by squeezing the lever allowing liquid nitrogen to cool the tip of the apparatus. The tip of the probe was placed on the tip of the ventricle for 5 s and then flushed with sterile saline with 1x penicillin/streptomycin to release the probe. A white ring of damaged tissue was observed that formed the new cryo-lesion. Probe size was adjusted and hold time optimized for animal size to create a lesion 1/3 of the ventricle, positioned at the tip. The pericardial sac was either sealed with vetbond (3 M) or ethilon silk sutures (for animals over 5 cm). The outer skin was then either sealed with vetbond (3 M) or ethilon silk sutures (for animals over 5 cm). Animals were recovered in clean 20% Holtfreters solution with antibiotics for 24 h (50 I.U./mL penicillin and 50 (μg/mL streptomycin) added to water for up to 7 days for Clo-Lipo and PBS-Lipo animals).

### Echocardiography analysis

Cardiac morphology and function at various stages post cryo-injury was analyzed using Vevo2100 ultrasound imaging system (FUJIFILM/VisualSonics Inc., Canada) with high frequency linear array transducer (VisualSonics MS550S, 32–56 MHz). Animals were lightly anaesthetized Tricane 0.05% and placed on the imaging platform in supine position supported by moistened tissues. Ultrasound transmission gel (Aquasonic, Parker laboratories, USA) was applied on the thorax. The transducer position was optimised to capture ventricular cross-sections in maximal longitudinal axis. B-mode cine-loop recordings of ventricle motion were acquired. All measurements and calculations were performed using Vevo^®^ 2100 software and compared using one-way ANOVA. %FAC was calculated as (Ventricular area; d—Ventricular area; s)/(Ventricular area; d) × 100. %FAC calculations gave similar results to independent measurements in LV-trace mode that allows for volumetric calculations (e.g., %EF and stroke volume). %FAC was chosen as a measurement to report heart function as LV-trace mode is optimized for the assessment of heart function in small mammals and in case of the axolotl, should be only regarded as an approximation. Hearts showing major vascular occlusion, anemia, rupture, or no clear lesion were eliminated from analysis and animals euthanized.

### Histology and immunohistochemistry

For histological and immunohistochemical analysis, tissue cryosections were prepared by either perfusing tissue with 0.7 × PBS (APBS) then fixation using freshly prepared 4% formaldehyde for 15 mins. The fixed tissues were submerged in 30% sucrose/PBS for 1 h followed by embedding with Optimal Cutting Temperature (OCT). Alternatively, samples were flash frozen in OCT and post-fixed with acetone or methanol to enhance staining. 18 μm thick sections were prepared before permeabilization with 0.1% Triton-X-100/PBS for 30 min. Sections were blocked with blocking solution (3% BSA, 1.5% normal donkey serum and 0.1% Triton-X-100 in PBS) and incubated overnight at 4 °C with primary antibodies (see antibody section). For histology, samples were fixed overnight in 4% paraformaldehyde in 0.7 × PBS at 4 °C, then rinsed, dehydrated, embedded in paraffin and sectioned at a thickness of 14 μm. Before staining, slides were de-paraffinized through three baths of xylene for 5 min each. Slides were then rehydrated in a graded series of 100, 90, 70, and 50% ethanol and then finally hydrated in distilled water for 5 min each. Sections were washed and counterstained with DAPI (Sigma) and mounted in Vectamount fluorescent mounting medium (Vector labs) or Mowbiol. Confocal microscopy was performed on a Nikon C1, Leica SP8 or Zeiss Axiovert 200 M microscope and images processes using Imaris software (Bitplane). Quantitation of immunofluorescence was done blinded using a Fiji (image J software).

### Cytochemistry

Reagents for Acid Fuchsin Orange G and Picosirius red staining were purchased from sigma and performed according to standard procedures (ihcworld.com). Picosirius red analysis was performed on a Leica DM4500 using a polarized filter set at maximal contrast.

### Antibodies

The following antibodies were purchased. Anti Nkx2.5: Abcam (ab35842), Anti BrdU: Becton Dickinson (B44), Troponin T: DSHB (CT3), Tenascin C: (DSHB), Anti-Fibronectin antibody: Abcam (ab23750), Anti-Collagen I: Millipore, Alpha SMA Anti-smooth muscle Actin: Invitrogen (1A4), DDR2: Santa Cruz (H-108), vWF Anti-von Willebrand factor: Sigma (F3520), IGF-I: Santa Cruz (H-70), IGF1-receptor beta antibody: Cell Signaling (3027 S), Anti-PCNA: Dako (PC10). Conjugated Isolectin B4 (IB4): Vectorlabs. Antibodies were titrated for specific application. All other secondary antibodies were purchased from Invitrogen (highly cross absorbed).

### RT-PCR gene expression analysis

RNA was isolated from harvested tissue using Trizol reagent (Invitrogen). Following isolation, RNA was further purified using the Zymopure RNA isolation kit with on column DNA digestion. RNA quality was assessed by spectrophotometry using a NanoDrop ND-1000 (NanoDrop, Wilmington, USA). Reverse transcription was performed using the Superscript VILO cDNA synthesis kit (Life technologies). Quantitative PCR assays were performed using LightCycler 480 SYBR green (Roche) and analyzed using a LC480 Thermal Cycler instrument (Roche). Gene expression levels were calculated using the 2^−ΔΔC^t and were normalized by using the geometric mean of at least three housekeeping (normalizer) genes. Experiments were performed at least three times and from three biological replicates. Primer sequences used in RT-PCR gene expression analysis are listed in Table S1.

### Statistical analysis

Data were shown throughout as mean values ± s.e.m. Analyses of significant differences between means were performed using two-tailed Student’s t-tests or 2-way ANOVA. With Turkeys multiple comparisons test. Alpha = 0.05. In all cases *P,0.05; **P,0.01., ***P,0.001, ****P,0.0001. NS = not significant.

### Data availability

All data are provided in the results section and supplementary information accompanying this paper.

## Electronic supplementary material

Supplementary Table 1

Supplementary figure 1

Supplementary figure 2
