# Supplementary material for: Heart regeneration in the salamander relies on macrophage-mediated control of fibroblast activation and the extracellular landscape
Source: NPJ Regen Med. 2017 Jul 27;2:22. doi: 10.1038/s41536-017-0027-y (PMC5677961; doi:10.1038/s41536-017-0027-y)
Supplement: Supplementary file 1 — Supplementary Table 1 [file 41536_2017_27_MOESM1_ESM.pdf]

Supplementary Table 1. Primer Sequences used for qPCR

Primer	Forward	Reverse
RBL27	CATCAGATCAAGCAAGCAGTA	CCAATGCAGCAGTTTAGATG
β-actin	TCCATGAAGGCTGCCCAACT	TGGCGCCACATCTGATTGAT
GAPDH	GACAAGGCATCTGCTCACCT	ATGTTCTGGTTGGCACCTCT
CSF1R	CTCCAGGATGGGACTGTCAT	CCGCTTGGAGGTAGAGTCTG
MPO	TCAACAGCTGGAGAATCGTG	ATGTTGATGGCGGCTAAATC
Collagen I-A2 (Col1a2)	ACCTGGTGGAAAGGGAGACT	GAAGATCCAGGTTCCCCATT
TGF- β1	CAAGCGTGCTCTGGATGT	GCAGTTCTTCTCCTGTGA
DDR2	ACTTTGGGATGAGTCGGAAC	ACTTGCCCAATAGGATGCTC
Vimentin	AACCTTGCAGAGACCGAAGA	GTAGTCGTTGGCGTCCTGTT
DPP4	TTACATGTGCCTTCCCACTG	TTTGAAATTCGGTGCTCTTG
ICAM1	AGCATAACGGCAAGATGGTC	GGATTTTCCCTCAATCAGCA
LOX	AAGACACGTCGTGCGATTAC	CACGTCCGTGATGTCTATCC
LOXL1	CTACATCCTGAAGGTGGTGGT	GCAGTTAGTGGTGGAGACGA
LOXL4	GTGCCGGTGTAAATACGATG	CCAGGTCTGATCTGTAGGCA
LOX5a	CACTGCTCACACTCAGGGTT	ACTTGGAAGGTTGGGTTCAC
MMP2	GGCGATGGTCAAGTTGTAAA	GTTGTGGAGCACCAGAGAAA
MMP3	CCATACAGGGCTTGAATGCCATT	TTGGGTCAGCATTGGTG
MMP9	GCATCGTAGGATTCTCCATCA	ACCAGTGAAGGCCGTTCCGAT
MMP13	AAGCCCTCATGTATCCCATC	GGATTCCTCGAGCCATAGAG
MMP14	TGGATAACTGAATGTGCGGA	GACGCTGACACTCAACCTCA
MMP19	AGAGGACCAAGAAGCTACGC	TGGAAAGATCTCAGGGCTTC
Tgm2	ATGGCTCCGTCAAGAAGACT	TTTCTTCATCCGATCCTTCC
Adh1a3	CAAAAGAACCTTCCCGACAA	GAGGCATCCATTCTTCTCCA
