# Supplementary material for: Heart regeneration in the salamander relies on macrophage-mediated control of fibroblast activation and the extracellular landscape
Source: NPJ Regen Med. 2017 Jul 27;2:22. doi: 10.1038/s41536-017-0027-y (PMC5677961; doi:10.1038/s41536-017-0027-y)
Supplement: Supplementary file 2 — Supplementary figure 1 [file 41536_2017_27_MOESM2_ESM.pdf]

A.

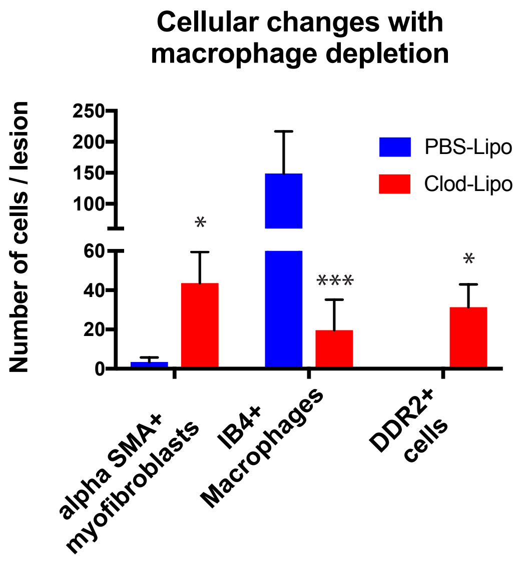


B.

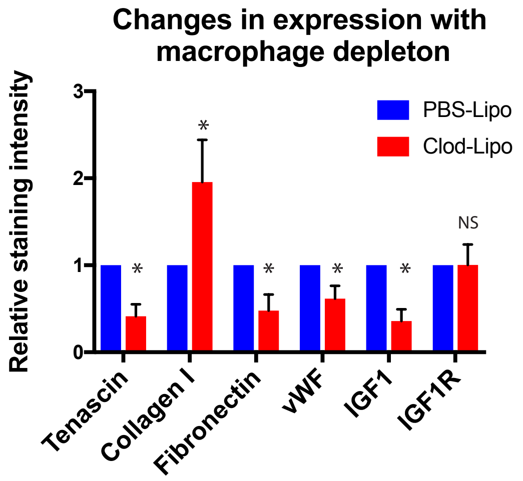


**Supplementary Figure 1.** Gene expression and cellular changes in the axolotl heart induced by macrophage depletion measured by immunofluorescent staining. Paired cardiac sections from both PBS-Lipo (Control) and Clod-Lipo (macrophage-depleted) animals14 days post cryo injury were stained in parallel. Equivalent tiled z-stack images were acquired using a Leica Sp8 confocal microscope under equivalent settings and conditions. Maximum projection images were produced in imaris to normalize focal planes. Quantitation of paired images was performed in Fiji (ImageJ) and expressed relative to PBS-Lipo control sections. Statistical analysis was performed using a two-tailed Student's t-tests. Alpha =0.05. Significance defined as *P,0.05; **P,0.01., ***P,0.001 and ns= not significant. This was performed for: **A.** Cellular changes with macrophage depletion. Alpha smooth muscle actin (alpha SMA) positive cells within the epicardium were not counted in this analysis, only cells within the 200 microns of the lesion were included. **B.** Changes in gene expression with macrophage depletion. The lesion was defined by the absence of troponin-T co-staining.
