# Supplementary material for: Heart regeneration in the salamander relies on macrophage-mediated control of fibroblast activation and the extracellular landscape
Source: NPJ Regen Med. 2017 Jul 27;2:22. doi: 10.1038/s41536-017-0027-y (PMC5677961; doi:10.1038/s41536-017-0027-y)
Supplement: Supplementary file 3 — Supplementary figure 2 [file 41536_2017_27_MOESM3_ESM.pdf]

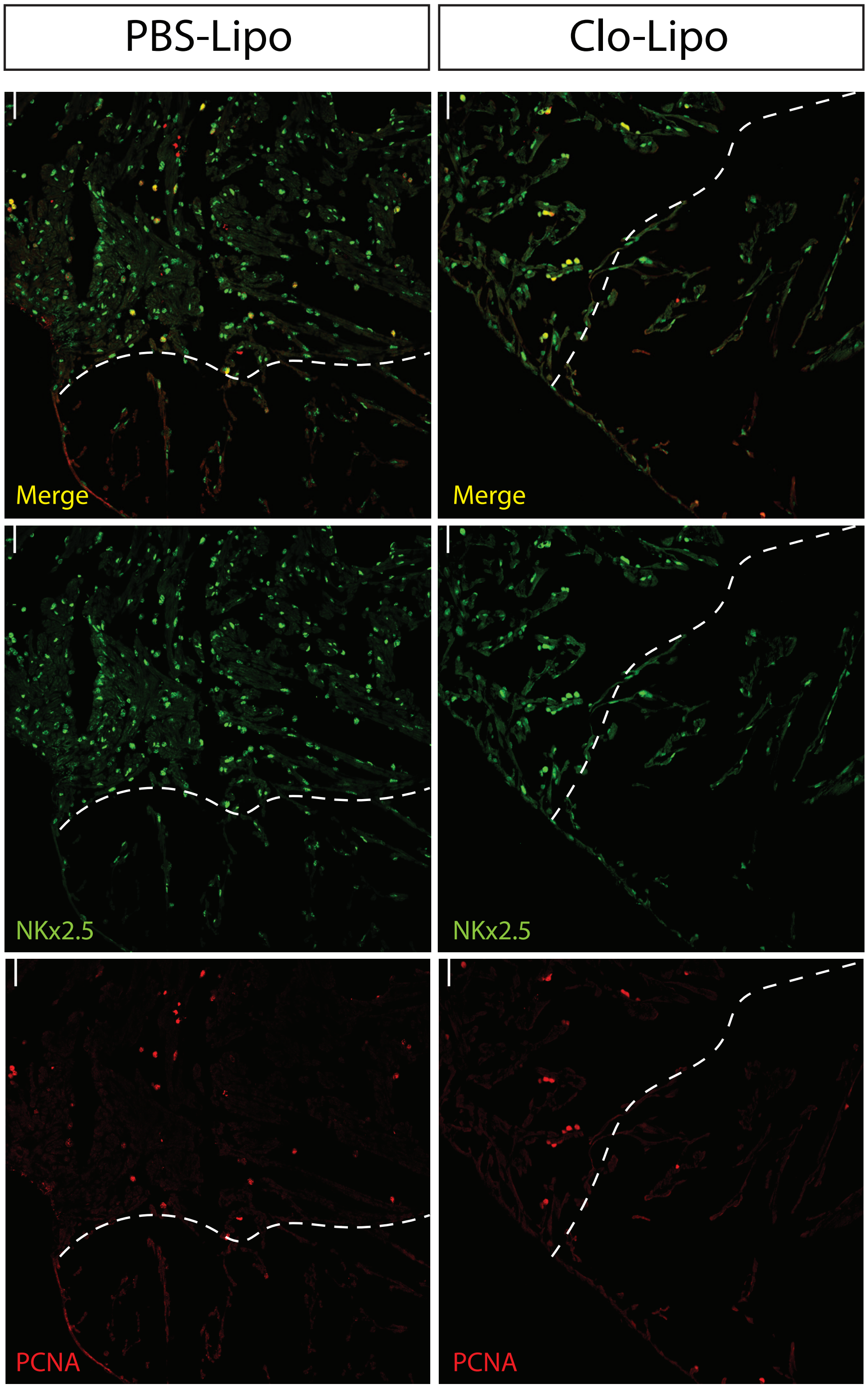

Supplementary figure 2. Staining strategy for counting CM proliferation in cryo injured hearts.
CMs were identified as PCNA+ (Red) Nkx2.5+ (Green) dual positive cells.
